# Should clinicians pay more attention to the potential underdiagnosis of osteoporosis in patients with ankylosing spondylitis? A national population-based study in Taiwan

**DOI:** 10.1371/journal.pone.0211835

**Published:** 2019-02-06

**Authors:** Li-Yu Hu, Ti Lu, Pan-Ming Chen, Cheng-Che Shen, Yao-Min Hung, Chiao-Lin Hsu

**Affiliations:** 1 Department of Psychiatry, Taipei Veterans General Hospital, Taipei, Taiwan; 2 Division of Psychiatry, Faculty of Medicine, National Yang-Ming University, Taipei, Taiwan; 3 Department of Social Work, Soochow University, Taipei, Taiwan; 4 Center for Geriatrics and Gerontology, Kaohsiung Veterans General Hospital; Kaohsiung, Taiwan; 5 Department of Psychiatry, Kaohsiung Veterans General Hospital, Kaohsiung, Taiwan; 6 Department of Psychiatry, Yuanshan Branch, Taipei Veterans General Hospital, Yilan, Taiwan; 7 Department of Information management, National Chung-Cheng University, Chiayi, Taiwan; 8 Department of Psychiatry, Taichung Veterans General Hospital, Chiayi Branch, Chiayi, Taiwan; 9 Department of Emergency Medicine, Kaohsiung Veterans General Hospital, Kaohsiung, Taiwan; 10 School of Medicine, National Yang Ming University, Taipei, Taiwan; 11 Center for Health Examination, Kaohsiung Veterans General Hospital, Kaohsiung, Taiwan; 12 Department of Nursing, Meiho University, Pingtung, Taiwan; 13 Department of Medical Education and Research and Research Center of Medical Informatics, Kaohsiung Veterans General Hospital, Kaohsiung, Taiwan; VU University Medical Center, NETHERLANDS

## Abstract

**Objectives:**

There are limited studies describing the association between ankylosing spondylitis (AS) and osteoporosis. We conducted a nationwide retrospective cohort study to investigate this epidemiologic evidence.

**Methods:**

Data were obtained from the Taiwan National Health Insurance Research Database (NHIRD). Of 10,290 participants, 2,058 patients with AS and 8,232 patients without AS were enrolled from the NHIRD between 2000 to 2013. Cumulative incidences of osteoporosis were compared between 2 groups. Cox regression model was used to estimate the hazard ratio (HR) of developing osteoporosis after controlling for demographic and other co-morbidities, and subgroup analyses were conducted to examine the risk factors for osteoporosis in AS patients.

**Results:**

The incidence rate ratio (IRR) of osteoporosis in AS patients was 2.17 times higher than that non-AS group (95% confidence interval [CI], 1.83–2.57). The adjusted HRs of osteoporosis for AS patients after controlling for demographic characteristics and comorbid medical disorders was 1.99 (95% CI 1.68–2.36). Among AS group, after adjustment for major comorbidities, old age (≥65 years, HR 4.32, 95% CI 3.01–6.18), female sex (HR 2.48, 95% CI 1.87–3.28), dyslipidemia (HR 1.44, 95% CI 1.01–2.06) were risk factors associated with osteoporosis.

**Conclusions:**

This cohort study demonstrated that patients with AS had a higher risk of developing osteoporosis, especially in those aged over 65, female sex and with dyslipidemia in this patient group.

## Introduction

Ankylosing spondylitis (AS) is a chronic inflammatory disease involving the sacroiliac joints and spine; in some cases, the peripheral joints, entheses, and/or extraarticular structures may also be involved [1]. AS has also been shown to be associated with other extra-articular inflammatory diseases including anterior uveitis, psoriasis, and chronic inflammatory bowel disease [2]. A previous systemic review demonstrated a varied prevalence of AS worldwide; 0.32% in North America, 0.24% in Europe, 0.17% in Asia, 0.10% in Latin America, and 0.07% in Africa [3]. The prevalence of AS in Taiwan is relatively low, between 0.19% and 0.4% [4]. Moreover, in general, AS is more common in males than in females [5].

Osteoporosis is considered to be a musculoskeletal disease identified by a decreased bone mineral density (BMD); the disease has also been shown to correlate with subsequent fragility fractures [6]. Evidence has shown that osteoporosis is relatively common in AS patients; the major pathophysiological mechanisms appear to be systemic inflammation and low BMD resulting from decreased daily physical activities caused by pain, stiffness, and/or ankyloses [7]. In addition, numerous studies have demonstrated that, in AS patients, a low BMD and bone loss are observed within the first 10 years of disease [8–10]. To summarize, studies have indicated that patients with AS are at increased risk of osteoporosis and subsequent osteoporotic fractures due to bone fragility [11].

Several studies have demonstrated that the prevalence of osteoporosis among AS patients varies widely, from 13% to 32% [8, 9, 12]. This wide variance is most likely due to the heterogeneity of the populations [8] or different BMD measurements [13]. Other studies have highlighted the possibility of underdiagnosis of osteoporosis among the AS population [14, 15]. In addition, few studies have explored the incidence of osteoporosis among AS patients, and those that have, are relatively small-scale (504 cases [16] and 17 cases [17]).

Therefore, our study aimed to investigate the association between AS and subsequent osteoporosis; we also sought to explore the potential risk factors and the incidence of new-onset osteoporosis among patients suffering from AS in Taiwan. From these aims, this study sought to strengthen previous small-scale studies and provide additional, clinically relevant, data for this issue. More importantly, we chose to investigate the possibility of underdiagnosis of osteoporosis among AS patients using the nationwide insurance database, which may be more reflective of real-world conditions; we also attempted to provide hypotheses to explain this condition based on both our data and the results of recent epidemiological studies.

## Materials and methods

### Data sources

The National Health Insurance (NHI) was started as a mandate by the Taiwanese government, with the coverage rate approaching 100% since March 1, 1995. The NHIRD is managed and released for research purposes publicly by the National Health Research Institutes (NHRI), with confidentiality being maintained according to the directives of the Bureau of the National Health Insurance (BNHI). This nationwide study from Taiwan’s NHIRD included all patients with a diagnosis of AS. This dataset included the diagnostic codes according to the International Classification of Diseases, Ninth Revision, Clinical Modification (ICD-9-CM). Database 2000 (LHID 2000) is a sub-dataset of the NHIRD with methodically and randomly screened-out the data in 2000 from the NHIRD; this includes all claim data of one million beneficiaries. In line with the Taiwanese NHRI reports, the distributions of age and sex, or the average insured payroll-related premiums, between the sample groups and all enrollees showed no significant differences [18, 19].

### Availability of data and materials section

The NHIRD is addressed in publicity by the NHRI and NHIRD is to be used only for research purposes. All applicants must obey the Computer-Processed Personal Data Protection Law [20] and the corresponding rules of the Bureaus of the NHI and NHRI. Moreover, applicants and their supervisors were asked for signed agreements upon application submission and all applications were reviewed for the approval of the data delivered. The application for the dataset may be mailed to the NHRI at nhird@nhri.org.tw or call at +886-037-246166 ext. 33603 for immediate assistance. Office hours: Monday–Friday, 8:00–17:30 (UTC+8).

The NHIRD, which was open to researchers in Taiwan, was available from the Health and Welfare Data Science Center (HWDC), Ministry of Health and Welfare (MOHW) (http://www.mohw.gov.tw/cht/DOS/). The data underlying this study was obtained from the NHIRD. Applicants interested in obtaining the data are able to propose a formal application to the Ministry of Health and Welfare of Taiwan. In the last sentence of the paragraph on the webpage, which states "Kindly visit MOHW and NHIA on-site services for NHI Data", the database was delivered to a higher-level government administration, called the "HWDC" for more efficient health-related data linkage, wider application, and better security management.

At present, interested researchers could still obtain the NHI data in Taiwan through a formal application to the Health and Welfare Data Science Center (HWDC), Department of Statistics, Ministry of Health and Welfare (MOHW). HWDC, MOHW website (currently only Chinese): http://dep.mohw.gov.tw/DOS/np-2497-113.html [21].

### Ethics statement

The NHI dataset consists of de-identified secondary data for research purposes, as such, the Institutional Review Board of Kaohsiung Veterans General Hospital issued a formal written waiver to obviate the need for consent. Therefore, in the present study, written consent was not obtained from the study participants. This study was authorized by the Institutional Review Board (IRB) of the Kaohsiung Veterans General Hospital as No. VGHKS14-CT7-07.

### Study population

**Fig 1:**
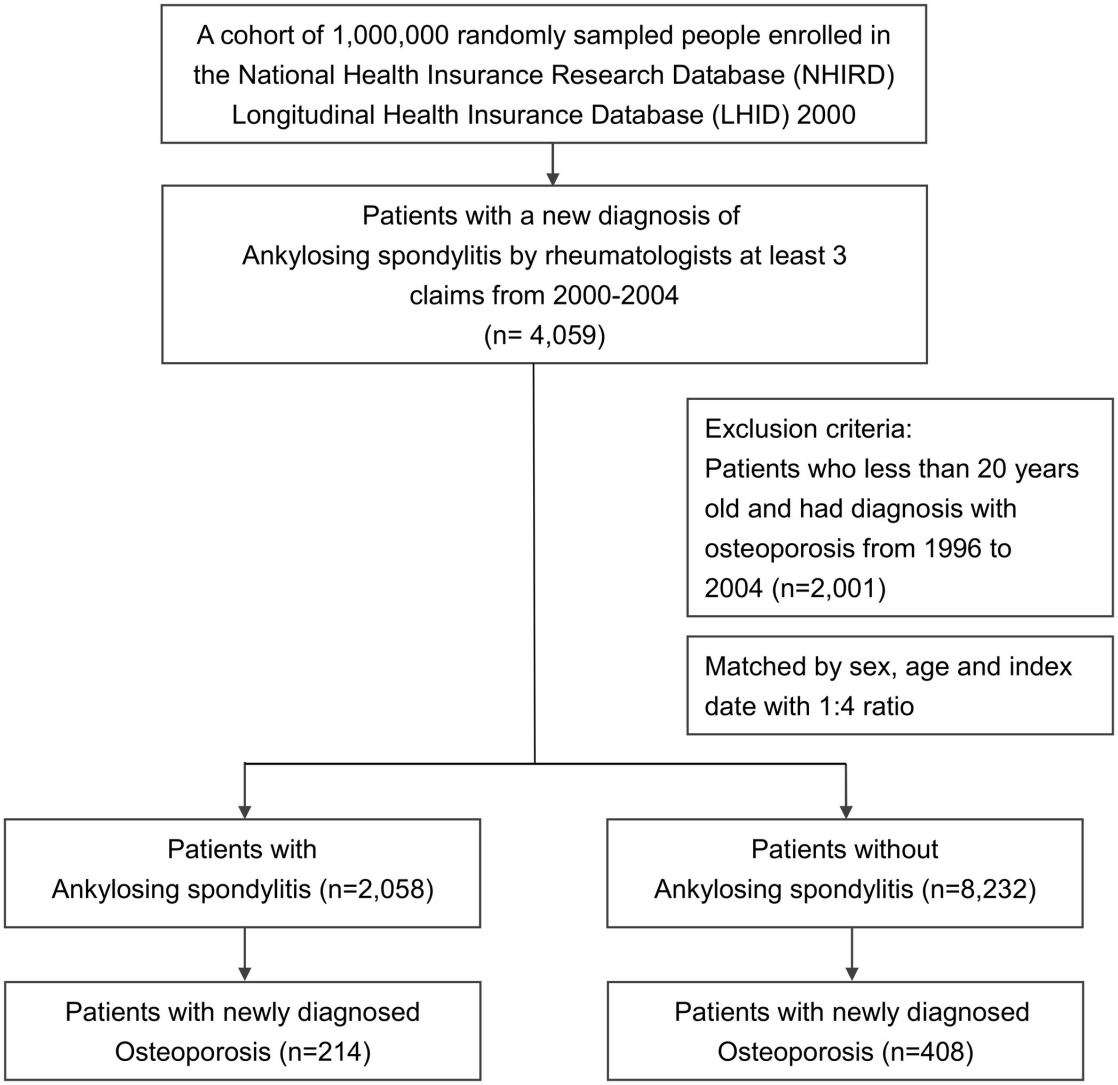
A flowchart demonstrating the enrollment of the study cohort. A total of 2,058 patient with history of Ankylosing spondylitis (AS) and 8,232 patients without history of AS were identified from Taiwan NHIRD. Among patients with and without the diagnosis of AS, 214 and 408 patients developed osteoporosis, respectively.

All patients aged 20 years and more, with a new diagnosis of AS (ICD-9-CM code 720.0) between January 1, 2000, and December 31, 2004, were enrolled. Only those who received a diagnosis of AS at least 3 times by a rheumatologist were selected. Since 1984, in Taiwan, the modified version of the New York criteria has been routinely used, in clinical practice and within the medical environment, as criteria for diagnosing patients with suspected AS [22]. We excluded patients who had been diagnosed with AS and osteoporosis (ICD-9-CM code 733.0 or 733.1) prior to the date of enrollment. Finally, we selected 8,232 participants with no history of AS, using the same exclusion criteria and within the same period, and ascertained that they were in accordance with the study cohort at a ratio of 1:4 in terms of age and gender (Fig 1).

All the ambulatory medical care and inpatient records for each subject in both groups were tracked from their index visit until December 31, 2013. The date of the first principal diagnosis of osteoporosis during the follow-up period was defined as the primary endpoint. Both cohorts were followed-up until osteoporosis was diagnosed, the death of the patient, withdrawal from the NHI programme, or until the end of 2013, whichever occurred first.

Several risk factors have been established in exploring the association between osteoporosis and other comorbidities [23–28] The variables analyzed in this study were as follows: age; sex; and relevant comorbidities including depressive disorder (ICD-9-CM codes, 296.2, 296.3, 300.4, and 311), hypertension (ICD-9-CM codes, 401–405), diabetes mellitus (DM)(ICD-9-CM code, 250), dyslipidemia (CD-9-CM code, 272), cerebrovascular disease (ICD-9-CM codes, 430–438), chronic obstructive pulmonary disease (COPD)(ICD-9-CM codes, 491, 492, and 496), nephropathy (ICD-9 CM codes, 580–589), autoimmune disease (ICD-9-CM codes, 136.1, 340, 443.1, 446.0–446.2, 446.4–446.7, 555, 556, 694.4, 710.0–710.4, and 714.0), obesity (ICD-9-CM code, 278.00), congestive heart failure (ICD-9-CM code, 428), hepatitis B (ICD-9-CM codes, 070.2 and 070.3), and hepatitis C (ICD-9-CM codes, 070.44, 070.54, and 070.7). All these variables were collected at the index date for the enrollment.

To investigate the urban-rural disparity, we adopted the urbanization stratification of Taiwan townships developed by Taiwan’s National Health Research Institutes. The level of urbanization was determined by the population density, the population ratio of individuals with higher educational levels, the population ratio of elderly individuals over 65 years-old, the population ratio of farmers, and the number of physicians per 100,000 people. The 368 townships in Taiwan were classified into 7 levels of urbanization, excluding the isolated isles in the Kingmen and Lienchiang counties. In the current study, we defined the level 1 and 2 townships as urban, level 3 and 4 townships as suburban, and level 5 to 7 townships, plus the isolated isles, as rural.

The monthly income was included as covariates in the multivariate model in order to calculate the adjusted HR. Insurance premiums, calculated according to the beneficiary’s total income, were used to estimate the monthly income. The monthly income was grouped into no income, low income (monthly income, 0 to 20,000 New Taiwan Dollar [NTD]), median income (monthly income, 20,000 NTD to 40,000 NTD), and high income (monthly income > 40,000 NTD).

### Statistical analysis

The incidence of newly diagnosed osteoporosis was calculated in both the AS and control groups, and they were stratified by sex and age (older or younger than 65 years). The variability, in terms of demographic characteristics, was compared between the AS and control groups by using independent t-tests, and chi-square tests when appropriate. The relevant period of person-years until an osteoporosis diagnosis was calculated from the enrollment date to the date of diagnosis of osteoporosis, death, withdrawal from the NHI system, or by the end of the follow-up (December 31, 2013).

A Cox proportional hazards regression model was applied to identify possible confounding factors in the univariate analysis and to exclude their effects on the evaluation of the AS-related risk of subsequent osteoporosis in the multivariate analysis. The variables used in the Cox model were as follows: age; sex; and common comorbidities including depressive disorder, hypertension, DM, dyslipidemia, cerebrovascular disease, COPD, nephropathy, autoimmune disease, hepatitis B, and hepatitis C. All variables that displayed a moderately significant statistical relationship with osteoporosis in the univariate analysis (P < 0.1) were included by forward selection in the multivariate analysis (Table 3). Moreover, we applied the Cox proportional hazards regression model to attempt to identify which variables may indicate subsequent osteoporosis among patients with AS (Table 4).

The incidence rate ratio (IRR) was calculated from the ratio between the two incidence densities (the rate in the AS group divided by the rate in the control group). To estimate the cumulative incidence of osteoporosis, we performed survival analysis of both cohorts using the Kaplan-Meier method and assessed the significance of the results using the log-rank test.

The data extraction, computation, linkage, processing, and sampling were performed using the SAS statistical software for Windows, Version 9.3 (SAS Institute, Cary, NC, USA), all other statistical analyses were performed using SPSS 20.0 for Windows (IBM, Armonk, NY, USA). The results of comparisons with a P-value of < 0.05 were considered to indicate a statistically significant relationship.

## Results

### Participant selection

The demographic and clinical variables between the AS and reference groups are presented in Table 1. According to the inclusion and exclusion criteria of this study, 2,058 patients with AS and 8,232 patients without AS were ultimately included in the outcome analysis. Among both cohorts, the median age at the enrollment date was 38 years (interquartile range [IQR], 28.3–50.1 years) and 63.8% were male. The median follow-up period was 10.87 years (IQR, 9.48–12.42 years) for AS patients and 11.03 years (IQR, 9.65–12.48 years) for non-AS patients. The frequency of comorbidities, including depressive disorder, hypertension, DM, dyslipidemia, cerebrovascular disease, COPD, nephropathy, autoimmune disease, obesity, congestive heart failure, hepatitis B, and hepatitis C, was higher in the AS cohort than the reference cohort. Furthermore, patients in the AS cohort had a significantly higher income than the reference group.

**Table 1:** Baseline characteristics of patients with and without ankylosing spondylitis.

Demographic data	Patients with AS *n* = 2,058		Patients without AS *n* = 8,232		P value
	*n*	%	*n*	%	
Age (years)^a^	38.01 (28.29–50.11)		38.01 (28.30–50.11)		
≥ 65	227	11.0	908	11.0	0.998
< 65	1,831	90.0	7,324	90.0	
Sex					
Male	1,314	63.8	5,256	63.8	0.999
Female	744	36.2	2,976	36.2	
Comorbidities					
Depressive disorder	44	2.1	66	0.8	< 0.001
Hypertension	428	20.8	1,300	15.8	< 0.001
Diabetes mellitus	234	11.4	709	8.6	< 0.001
Dyslipidemia	333	16.2	907	11.0	< 0.001
Cerebrovascular disease	277	13.5	672	8.2	< 0.001
COPD	227	11.0	632	7.7	< 0.001
Nephropathy	241	11.7	538	6.5	< 0.001
Autoimmune disease	76	3.7	163	2.0	< 0.001
Obesity	14	0.7	30	0.4	0.043
Congestive heart failure	44	2.1	102	1.2	0.003
Hepatitis B	80	3.9	158	1.9	< 0.001
Hepatitis C	17	0.8	34	0.4	0.022
Degree of urbanization					0.541
Urban	1,253	60.9	5,096	61.9	
Suburban	665	32.3	2,623	31.9	
Rural	140	6.8	513	6.2	
Income group					< 0.001
High income	340	16.5	1,077	13.1	
Medium income	416	20.2	1,687	20.5	
Low income	938	45.6	3,854	46.8	
No income	364	17.7	1,614	19.6	
Follow-up years^a^	10.87 (9.48–12.42)		11.03 (9.65–12.48)		< 0.001

^a^Median (interquartile range); COPD indicates chronic obstructive pulmonary disease

### The incidence of osteoporosis development

Among the AS group, 214 (10.21 per 1,000 person-years) patients developed osteoporosis during the study period. The incidence rate (IRR) was 2.17 (95% confidence interval [CI], 1.83–2.57, P < 0.001) in the AS group compared to that in the reference group. When stratified by sex and age, the IRR of osteoporosis in the current study remained higher in patients with AS than in the control patients; this was also the case among the male, or younger population (Table 2).

**Table 2:** Incidence of osteoporosis in patients with and without ankylosing spondylitis (AS).

	Patients with AS		Patients without AS		Rate ratio (95% CI)	P value
	No. of osteoporosis	Per 1,000 person-years	No. of osteoporosis	Per 1,000 person-years		
Total	214	10.21	408	4.70	2.17 (1.83–2.57)	< 0.001
Age						
≥ 65	77	53.36	164	23.34	2.29 (1.72–3.02)	< 0.001
< 65	137	7.02	244	3.06	2.29 (1.85–2.84)	< 0.001
Sex						
Male	88	6.51	133	2.38	2.73 (2.06–3.60)	< 0.001
Female	126	16.93	275	8.89	1.91 (1.53–2.36)	< 0.001
Follow-up						
0–1	55	1747.14	59	747.21	2.34 (1.59–3.43)	< 0.001
1–5	69	200.99	154	128.76	1.56 (1.16–2.09)	0.003
5–10	67	15.09	143	7.90	1.91 (1.41–2.57)	< 0.001
≥ 10	23	1.43	52	0.77	1.85 (1.08–3.07)	0.019

CI indicates confidence interval

**Fig 2:**
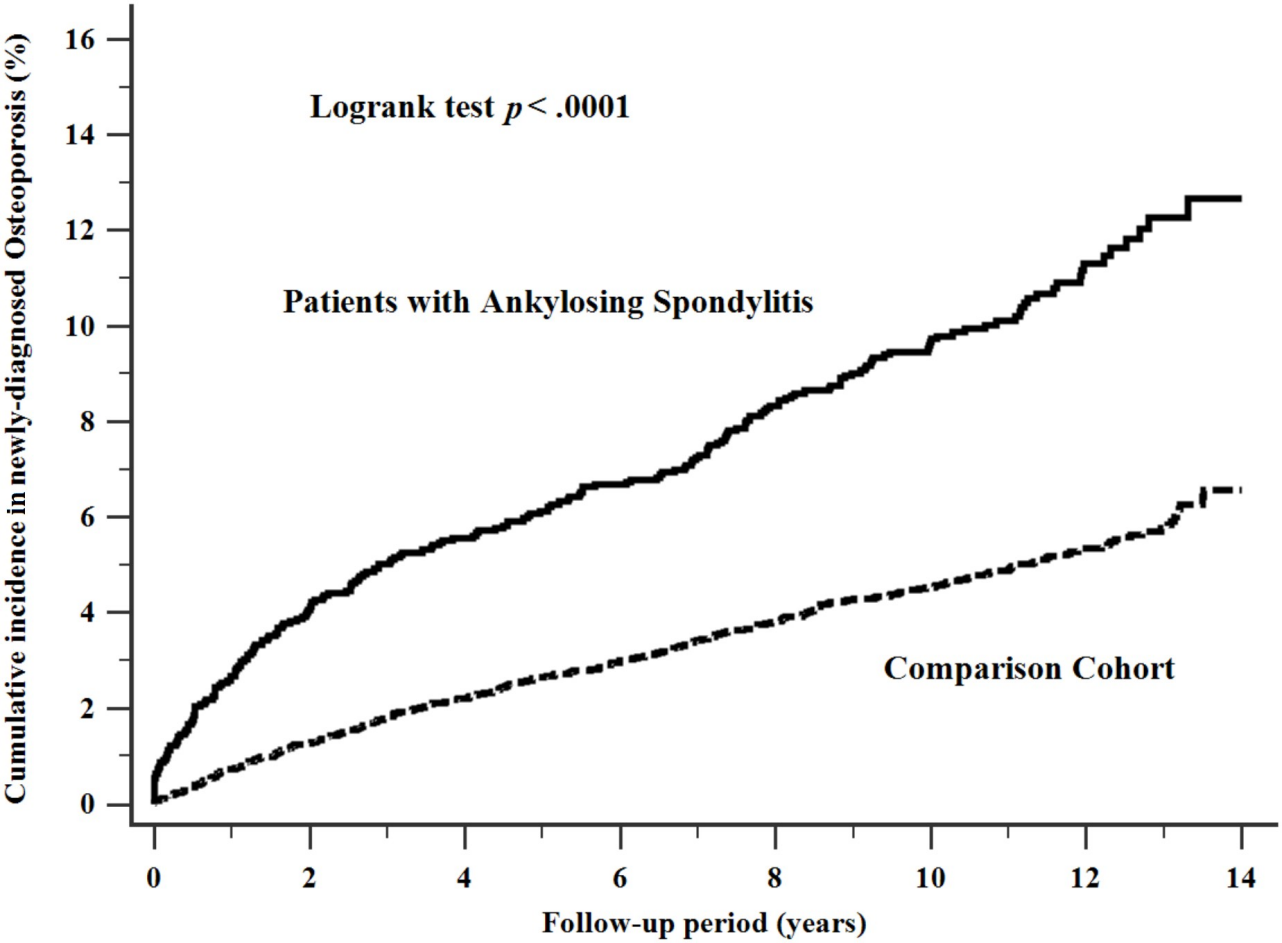
Cumulative incidence of osteoporosis in patients with ankylosing spondylitis. The cumulative incidence of newly diagnosed osteoporosis in patients with Ankylosing spondylitis was higher than the comparison cohort significantly.

The cumulative incidence of osteoporosis in patients with AS was 10.21 per 1000 person-years, which was higher than the comparison cohort (4.7 per 1000 person-years) during the follow-up period (log-rank test, P < 0.0001, as shown in Fig 2).

### The effects of AS on the risk of developing subsequent osteoporosis

As shown in Table 3, after adjusting for age, sex, the diagnosis of AS and comorbidities, we found that old age, female sex, hypertension, dyslipidemia, COPD and autoimmune disease history were independent risk factors of osteoporosis. The hazard ratio (HR) of the diagnosis of AS, old age, female sex, hypertension, dyslipidemia, COPD and autoimmune disease history was 2.17, 4.56, 3.39, 1.43, 1.43, 1.53 and 1.76, respectively, as compared with those of patients without osteoporosis.

**Table 3:** Analyses of risk factors for osteoporosis in patients with and without ankylosing spondylitis.

Predictive variables	Univariate analysis		Multivariate analysis	
	HR (95% CI)	P value	HR (95% CI)	P value
Ankylosing Spondylitis	2.17 (1.83–2.55)	< 0.001	1.99 (1.68–2.36)	< 0.001
Age (< 65 = 0, ≥ 65 = 1)	7.17 (6.09–8.43)	< 0.001	4.56 (3.70–5.61)	< 0.001
Sex (Male = 0, Female = 1)	3.27 (2.77–3.85)	< 0.001	3.39 (2.87–4.02)	< 0.001
Comorbidities				
Depressive disorder	1.11 (0.53–2.34)	0.781		
Hypertension	4.08 (3.48–4.79)	< 0.001	1.43 (1.16–1.76)	0.001
Diabetes mellitus	3.11 (2.57–3.78)	< 0.001	1.20 (0.95–1.50)	0.124
Dyslipidemia	3.13 (2.62–3.73)	< 0.001	1.43 (1.16–1.77)	0.001
Cerebrovascular disease	3.64 (2.96–4.49)	< 0.001	1.24 (0.98–1.56)	0.074
COPD	3.10 (2.53–3.78)	< 0.001	1.53 (1.23–1.90)	< 0.001
Nephropathy	2.38 (1.90–2.97)	< 0.001	1.06 (0.84–1.35)	0.621
Autoimmune disease	2.27 (1.72–3.02)	< 0.001	1.76 (1.32–2.34)	< 0.001
Hepatitis B	0.63 (0.33–1.22)	0.168		
Hepatitis C	2.76 (1.31–5.81)	0.008	1.44 (0.67–3.09)	0.349
Degree of urbanization				
Urban	Reference			
Suburban	1.41 (1.19–1.67)	0.003	1.12 (0.94–1.33)	0.205
Rural	2.16 (1.66–2.81)	< 0.001	1.19 (0.90–1.56)	0.222
Income group				
High income	Reference		Reference	
Medium income	1.12 (0.78–1.62)	0.544	0.96 (0.67–1.39)	0.836
Low income	2.42 (1.78–3.29)	< 0.001	1.33 (0.97–1.83)	0.082
No income	2.64 (1.90–3.67)	0.378	1.10 (0.78–1.56)	0.585

HR, hazard ratio; CI, confidence interval; COPD, chronic obstructive pulmonary disease

### Risk factors of developing osteoporosis in AS patients

Univariate analysis was used to identify the possible risk factors for subsequent osteoporosis in the AS cohort, with the results demonstrated in Table 4. We included these potential confounding variables in a Cox regression model for multivariate analysis. The results of this subgroup analysis showed that old age (≥ 65 years old) (HR = 4.32, 95% CI, 3.01–6.18, P < 0.001), female sex (HR = 2.48, 95% CI, 1.87–3.28, P < 0.001), dyslipidemia (HR = 1.44, 95% CI, 1.01–2.06, P = 0.044) were risk factors in AS patients; these results are consistent with the risk factors within the general population. In addition, living in a suburban area (HR = 1.43, 95% CI, 1.06–1.92, P = 0.018) was an independent risk factor for subsequent osteoporosis development in patients with AS. Furthermore, those with a history of COPD were also shown to have a marginal correlation with a higher risk of developing osteoporosis (HR = 1.43, 95% CI, 0.98–2.08, P = 0.064).

**Table 4:** Analyses of risk factors for osteoporosis in patients with ankylosing spondylitis.

Predictive variables	Univariate analysis		Multivariate analysis	
	HR (95% CI)	P value	HR (95% CI)	P value
Age (< 65 = 0, ≥ 65 = 1)	7.00 (5.27–9.28)	< 0.001	4.32 (3.01–6.18)	< 0.001
Sex (Male = 0, Female = 1)	2.59 (1.97–3.39)	< 0.001	2.48 (1.87–3.28)	< 0.001
Comorbidities				
Depressive disorder	1.37 (0.61–3.09)	0.446		
Hypertension	2.98 (2.26–3.92)	< 0.001	1.10 (0.77–1.57)	0.595
Diabetes mellitus	2.73 (1.99–3.76)	< 0.001	1.33 (0.91–1.95)	0.144
Dyslipidemia	2.67 (2.01–3.57)	< 0.001	1.44 (1.01–2.06)	0.044
Cerebrovascular disease	2.82 (2.00–3.96)	< 0.001	1.15 (0.78–1.70)	0.478
COPD	2.12 (1.50–3.00)	< 0.001	1.43 (0.98–2.08)	0.064
Nephropathy	2.06 (1.47–2.87)	< 0.001	1.13 (0.79–1.62)	0.513
Autoimmune disease	1.46 (0.95–2.23)	0.083	1.41 (0.91–2.18)	0.120
Hepatitis B	0.58 (0.24–1.42)	0.233		
Hepatitis C	2.75 (1.02–7.40)	0.045	1.72 (0.61–4.84)	0.302
Degree of urbanization				
Urban	Reference		Reference	
Suburban	2.03 (1.53–2.68)	< 0.001	1.43 (1.06–1.92)	0.018
Rural	2.03 (1.25–3.28)	0.004	1.05 (0.63–1.75)	0.851
Income group				
High income	Reference		Reference	
Medium income	0.31 (0.17–0.54)	< 0.001	1.09 (0.58–2.06)	0.780
Low income	0.40 (0.25–0.65)	< 0.001	1.67 (0.97–2.87)	0.064
No income	0.94 (0.67–1.30)	0.697	1.74 (0.98–3.11)	0.060

HR, hazard ratio; CI, confidence interval; COPD, chronic obstructive pulmonary disease

## Discussion

Since the NHIRD serves patients from all areas in Taiwan, these patients are sufficiently representative of the nation as a whole. Our study explored the incidence of newly diagnosed osteoporosis in patients with and without AS in Asia. There are two major findings of this study. First, the risk of osteoporosis is significantly higher among patients with AS. Second, we suggest that old age (≥ 65 years old), female sex, and dyslipidemia may be considered as potential risk factors for developing subsequent osteoporosis among patients with AS.

From previous studies, it is well-recognized that AS patients have higher risk of osteoporosis [29]. However, in the present study, the incidence of osteoporosis in AS patients within 1 year of diagnosis was 2.67% (55/2058 = 2.67%); this is much lower than has been previously shown in the Netherlands (9%) [7], China (9.7%) [16], Morocco (25%) [9], and Germany (40.7%) [13]. The difference in follow-up period, as well as differences in geographical region and sex disparity may contribute to this variability.

A current United States cohort study using the registered database and including 46,265 patients with AS, demonstrated that AS patients are significantly more likely to develop osteoporosis than non-AS patients; these results agree with the findings of the present study [30]. However, the incidence rate of osteoporosis among the AS group in the current study was lower than the cohort conducted in the United States (10.21/1,000 person-years vs. 22.6/1,000 person-years); this may be due to the younger population in our study (38 years old vs. 51 years old), the male/female ratio, genetic heterogeneity, and the duration of disease.

We speculated that the number of male AS patients with subsequent osteoporosis is underrecognized and underestimated in our database. There are several potential reasons for this underestimation. First, AS-related syndesmophytes falsely increase the BMD measured by dual-energy x-ray absorptiometry (DXA) [31]. Second, patients with AS usually don’t seek medical treatment unless severe symptoms occur; this, in turn, may result in low DXA or X-ray utilization and thus fewer opportunities for a diagnosis of osteoporosis. One study supports this speculation, reporting that only patients with the most severe vertebral fracture will seek medical advice [32]. Third, clinicians may overlook osteoporosis due to the aim of the initial treatment frequently being directed at the control of symptoms [29]. Fourth, osteoporosis is not usually suspected in a young-male dominant patient group and young men are less likely to be screened according to practice guidelines [33, 34]. Lastly, the unusual results may be due to the fact that many patients and/or physicians are unaware of the increased risk of osteoporosis among the relatively young, and predominantly male, population [8, 29].

AS is recognized as a disease of young men and osteoporosis is usually not suspected in this demographic. Therefore, it would be reasonable to expect most AS patients with subsequent osteoporosis to be male and young. However, our results were not in line with these expectations. We found that old age (≥ 65 years old), female sex, and dyslipidemia are potential risk factors for developing osteoporosis among patients with AS. In the general population, older females, especially postmenopausal females, are well-known to have an increased risk of osteoporosis; this is also the case in AS patients, as has been reported in an earlier study [15].

Our results also suggested that dyslipidemia is a risk factor for developing osteoporosis in AS patients. The link between cardiovascular disease [35], or serum lipids, and low BMD/osteoporosis has been investigated in several studies [36–38]. Osteoporosis and cardiovascular disease share numerous common risk factors, including age and serum oxidized lipid [39]. The underlying mechanisms are thought to originate from a distorted lipid metabolism resulting in higher serum oxidized lipids, which, in turn, suppress osteoblast differentiation [40]. As demonstrated by a murine study by Graham at el al., oxidized lipids will adversely affect bone mechanical strength and impair bone regeneration by inhibiting the differentiation of osteoblasts and enhancing the presence of osteoclasts [41]. Additionally, adipocytokines secreted by adipose cells may enhance osteoclast formation, and oxidized LDL may suppress osteoblast formation [42]. Second, oxidized lipids impair angiogenesis and blood vessel functioning, both of which lead to decreased osteogenesis [43]. The accumulation of oxidized lipids in the atherosclerotic plaque within bone blood vessels will increase the levels of anti-osteoblastogenic inflammatory cytokines and decrease the pro-osteoblastogenic effect [44–46].

Our data demonstrate that the risk of developing osteoporosis is marginally higher in patients with a history of COPD, which is consistent with previous studies [47–49]. The present study also identified a higher risk of osteoporosis in AS patients who live in suburban areas; a lower 25-hydroxy-vitamin D level among the low socio-economic population is one of the possible reasons for this increased risk [50, 51].

The strengths of our study include the large sample size and the fact that we attempted to ensure a high diagnostic accuracy of AS by only obtaining coding from a rheumatologist. Since 1984, modified version of the New York criteria is used routinely in clinical practice in Taiwan as criteria for diagnosing patients with suspected AS. We believe that the diagnosis of AS based on clinical coding by a rheumatologist is reliable in the majority of cases and that the long observation period of the present study offers the opportunity to evaluate the real impact of AS. The national insurance system database is also representative of real conditions in clinical settings. Furthermore, the NHIRD could track each guaranteed case from different medical institutes over a period of time to avoid a dropping-out bias and to minimize the possibility of recall bias.

However, certain limitations in the present study should be taken into consideration. First, personal information related to osteoporosis, such as smoking history, alcohol consumption, lifelong steroid use, low body mass index, lab data including serum inflammation markers, calcium level, vitamin D intake, and serum uric acid [52], may have influenced our results. Although it is widely known that smoking increases the risk of osteoporosis, we attempted to use COPD as a proxy variable for cigarette smoking, which has been accepted in several previous studies [53–55]. Second, details relevant to the disease severity, and those related to the disease activity of AS, are somewhat lacking in the claims data; thus, in the present study, we could not evaluate the impact of the severity and disease activity of AS on osteoporosis. Third, our study did not provide any information about medications administered, which may interfere with the BMD. Finally, we should be aware of some possible investigation bias. For example, patients who were not followed-up adequately and/or perhaps did not receive complete treatment due to a particular disease, could raise the possibility of other subclinical diseases being present within the study population; therefore, the incidence of osteoporosis within the control group may be underestimated.

## Conclusions

Although AS is a risk factor for the development of new-onset osteoporosis, there are no existing guidelines to routinely assess patients with AS for osteoporosis, and young men are less likely to be screened. Given that the underestimation of osteoporosis in AS patients can have serious health consequences, a greater emphasis should be placed on this association. This population-based cohort study demonstrated that patients with AS have a higher risk of developing osteoporosis, especially in those aged over 65 years, of female sex, and with dyslipidemia.

## Abbreviations

AS: Ankylosing spondylitis
BMD: bone mineral density
BNHI: Bureau of the National Health Insurance
COPD: chronic obstructive pulmonary disease
DM: diabetes mellitus
HWDC: Health and Welfare Data Science Center
ICD-9-CM: International Classification of Diseases, Ninth Revision, Clinical Modification
LHID: Longitudinal Health Insurance Database
MOHW: Ministry of Health and Welfare
NHI: National Health Insurance
NHIRD: National Health Insurance Research Database
NTD: New Taiwan Dollar
